# Monthly Record of Deaths in the City of Buffalo

**Published:** 1858-11

**Authors:** H. D. Garvin

**Affiliations:** Health Physician


ART. VIII.—*Report of Mortality in Buffalo for the Month of Sept.*, 1858.
By H. D. GARVIN, M. D., Health Physician.

| DISEASES. | No. | Males. | Females. | No Sex given. |
|---|---|---|---|---|
| Abcess, | 1 | | 1 | |
| Accidental, | 8 | 7 | 1 | |
| Apoplexy, | 1 | 1 | | |
| Cancer of Stomach, | 1 | | 1 | |
| Cerebral Disease, | 1 | 1 | | |
| Chicken Pox, | 1 | 1 | | |
| Cholera Infantum, | 49 | 27 | 16 | 6 |
| Congestion of Brain, | 3 | 3 | | |
| " " Lungs, | 2 | 2 | | |
| Convulsions, | 10 | 4 | 3 | 3 |
| Croup, | 1 | 1 | | |
| Delirium Tremens, | 3 | 3 | | |
| Diarrhœa, | 30 | 16 | 10 | 4 |
| Dysentery, | 27 | 17 | 7 | 3 |
| Enteritis, | 1 | | 1 | |
| Erysipelas, | 1 | 1 | | |
| Fever, Typhoid, | 8 | 2 | 6 | |
| " Typhus, | 1 | 1 | | |
| " Puerperal, | 1 | | 1 | |
| " Remittent, | 1 | | 1 | |
| " Nervous, | 1 | | 1 | |
| Hooping Cough, | 5 | 4 | 1 | |
| Hepatic Disease, | 1 | 1 | | |
| Hydrocephalus, | 5 | 4 | 1 | |
| Intemperance, | 2 | 1 | 1 | |
| Jaundice, | 1 | | 1 | |
| Marasmus, | 20 | 11 | 6 | 3 |
| Meningitis, | 1 | | 1 | |
| Old Age, | 3 | 2 | 1 | |
| Paralysis, | 1 | 1 | | |
| Peripneumonia Notha, | 1 | | 1 | |
| Peritonitis, | 2 | 2 | | |
| Phthisis Pulmonalis, | 26 | 12 | 10 | 4 |
| Premature Birth, | 1 | 1 | | |
| Pulmonary Apoplexy, | 1 | 1 | | |
| Still Born, | 9 | 4 | 3 | 2 |
| Scrofula, | 2 | 1 | 1 | |
| Tabes Mesenterica, | 1 | 1 | | |
| Teething, | 2 | 1 | 1 | |
| Typhoid Dysentery, | 1 | | 1 | |
| Tpyhus Abd., | 4 | | 1 | |
| Uterine Disease, | 2 | | 2 | |
| Unknown, | 16 | | | |
| Total, | 256 | | | |

SEXES.

| | |
|---|---|
| Males, | 134 |
| Females, | 81 |
| Sex not given, | 25 |
| Total, | 256 |

AGES.

| | | | |
|---|---|---|---|
| Still-born, | 9 | Between 20 years and 30 years, | 15 |
| 1 day, | 0 | " 30 " " 40 " | 21 |
| 1 day and 30 days, | 15 | " 40 " " 50 " | 9 |
| Between 1 month and 6 months, | 38 | " 50 " " 60 " | 7 |
| " 6 months and 12 " | 37 | " 60 " " 70 " | 4 |
| " 1 year " 3 years, | 60 | " 70 " " 80 " | 2 |
| " 3 " " 5 " | 9 | " 80 " " 90 " | 1 |
| " 5 " " 10 " | 10 | " 90 " " 100 " | 0 |
| " 10 " " 20 " | 4 | " 100 " | 0 |
| | 182 | | 59 |
| Ages not given, | 14 | | 241 |
| Total, | 256 | | |

NATIVITIES.

| | | | |
|---|---|---|---|
| American, | 192 | Prussian, | 0 |
| German, | 27 | Italian, | 0 |
| Irish, | 18 | French, | 1 |
| English, | 3 | Scotch, | 0 |
| Canadian, | 2 | Switzerland, | 0 |
| Wales, | 0 | Country not given, | 13 |
| Total, | | | 256 |

---

*Physiology, Pathology and Therapeutics of Muscular Exercise. A Paper read before the Cook County Medical Society, and Published at their Request.* By W. H. Byford, M. D., Professor of Obstetrics and Diseases of Women and Children in Rush Medical College.

The above is a well written paper of twenty-six pages, in which muscular exercise is considered in the points of view indicated by the title. The subject is discussed in an able and satisfactory manner, and it is shown that this means of treatment in disease is of great importance. The well as well as the sick, should give attention to muscular exercise; for every one knows that a want of attention to this point is the cause of many of the chronic ailments which we are called upon to treat. We are glad to see the attention of the profession directed particularly to this subject, and hope that the practice of taking muscular exercise into account in the treatment of disease may become more general.

